# Historical Perspective of HER2 Testing and Treatment in Prostate Cancer

**DOI:** 10.3390/curroncol33020091

**Published:** 2026-02-02

**Authors:** Natalia Zamalloa, Jacqueline Rose, Coen J. Lap, Rithika Rajendran, Fayez Estephan, Karan Jatwani, Aarati Poudel, Ramesh Subrahmanyam, Paula J. Hurley, Victor E. Nava, Maneesh Jain

**Affiliations:** 1 Department of Pathology, George Washington University, Washington, DC 20037, USA; 2 Department of Pathology and Laboratory Medical Service, Veterans Affairs Medical Center, Washington, DC 20422, USA; 3 Department of Internal Medicine, George Washington University, Washington, DC 20037, USA; 4 Section of Hematology and Oncology, Edward P. Evans Precision Oncology Center of Excellence, Veterans Affairs Medical Center, Washington, DC 20422, USA; 5 Lombardi Comprehensive Cancer Center, Medstar Georgetown University Hospital, Washington, DC 20007, USA; 6 Department of Internal Medicine, Capital Health Regional Medical Center, Trenton, NJ 08638, USA; 7 Department of Hematology and Oncology, George Washington University, Washington, DC 20037, USA; 8 Department of Medicine, Vanderbilt University Medical Center, Nashville, TN 37232, USA

**Keywords:** prostate cancer, biomarkers, therapeutic targets, quality of life, immunohistochemistry, antibody–drug conjugate

## Abstract

Human epidermal growth factor receptor 2 (HER2) is a molecular target of interest in prostate cancer due to its association with poor prognosis and its potential role in androgen receptor signaling. However, earlier clinical trials investigating HER2-targeted therapies, including antibodies and small molecules, have shown limited efficacy. More recent studies using the HER2 antibody-drug conjugate (ADC) trastuzumab deruxtecan (T-DXd) suggest potential therapeutic benefit in prostate cancer. However, its effective utilization requires a HER2 IHC scoring system that accurately represents HER2 expression patterns unique to prostate cancer, which is currently not established. We have developed a modified HER2 IHC scoring system that, unlike the breast and gastrointestinal tumor HER2 IHC grading scales, considers the distinct spatiotemporal expression of HER2 in prostate tumors. In this commentary, we discussed two patients with metastatic prostate cancer who were classified as HER2 IHC 3+ using our prostate cancer-specific scoring system and who demonstrated meaningful clinical responses and responded to treatment with T-DXd. We further review the historical evolution of HER2 testing in prostate cancer, as well as factors that may have contributed to the failure of previous clinical trials targeting HER2 in prostate tumors. Our aim is to highlight the need for developing a standardized HER2 IHC grading model in prostate cancer, which could improve the predictive value of HER2 IHC expression, enabling a more accurate identification of patients likely to benefit from HER2-targeted ADCs.

## Simple Summary

A commentary examining the historical significance of human epidermal growth factor receptor 2 (HER2) immunohistochemistry (IHC) in prostate cancer, summarizing the early clinical trials related to HER2-positive prostate cancer, showcasing new trials using HER2 antibody-drug conjugate (ADC) transtuzumab deruxtecan (T-DXd), underscoring the need for a prostate-specific HER2 IHC scoring system, proposing a scoring system in comparison with the current Breast and Gastroesophageal Junction HER-2 scoring system and possible future targeted therapy in HER2-positive prostate cancer. We aim to highlight the need for a Prostate Cancer specific HER2 scoring system.

## 1. Introduction

Prostate cancer is the most common cancer in men in the United States [1]. Despite substantial advances in prostate cancer detection and management, continued efforts are needed to develop more precise and individualized therapeutic approaches. Human epidermal growth factor receptor 2 (HER2) is a target of interest in prostate cancer due to its role in the activation of androgen receptor signaling [2]. Additionally, HER2 expression in prostate cancer is associated with more aggressive disease and poor prognosis [3]. Together, these findings underscore the need to explore clinically meaningful HER2-targeted treatments for prostate cancer. In this commentary, we aim to review the historical significance of HER2 immunohistochemistry (IHC) in prostate cancer, discuss previous clinical trials targeting HER2 in prostate cancer, and highlight the need for a prostate-specific HER2 scoring system which could improve the predictive value of HER2 IHC expression, enabling a more accurate identification of patients likely to benefit from HER2-targeted antibody–drug conjugates (ADCs).

## 2. History of HER2 Testing in Prostate Cancer

The evolution of HER2 testing in prostate cancer has influenced our current understanding of its biological and therapeutic significance. Preliminary investigations about the role of HER2 in prostate cancer in the 1990s relied solely on immunohistochemistry (IHC) using non-standardized antibodies, staining techniques, and fixation protocols, which resulted in highly variable HER2 expression rates in prostate cancer tissue [4]. The introduction of fluorescence in situ hybridization (FISH) provided a more reliable means of evaluating *HER2* gene alterations and initially suggested a positive correlation between gene amplification and disease stage [5]. Importantly, these early genomic analyses did not utilize standardized criteria for gene amplification and likely overestimated its role in HER2 protein overexpression in prostate cancer [6]. Subsequent studies that combined IHC, ribonucleic acid in situ hybridization, and FISH further refined these observations, suggesting that *HER2* upregulation in prostate cancer likely occurs predominantly at the transcriptional level without evidence of significant gene amplification [7]. More advanced HER2 detection techniques, such as high-throughput tissue microarray (TMA) analysis, confirmed that *HER2* gene amplification is rare and may pose no correlation with HER2 protein expression in prostate cancer. This may partially explain why early HER2-targeted therapies failed in treating prostate cancer, given that they were also ineffective against breast cancers with low gene amplification [6]. More recent genomic profiling studies using next-generation sequencing (NGS) have reaffirmed the rarity of *HER2* gene amplification in prostate tumors, which leave protein-based testing, such as IHC, as the primary modality for HER2 detection [8,9]. These methodological refinements have clarified earlier inconsistencies in HER2 expression and informed the interpretation of therapeutic failures involving HER2-targeted agents.

## 3. Previous Clinical Trials Targeting HER2 in Prostate Cancer

Early HER2-targeted clinical trials failed to show meaningful therapeutic benefit in prostate cancer. Trastuzumab (Herceptin), a HER2-directed monoclonal antibody and inhibitor of downstream HER2 signaling, failed to elicit a significant clinical response both as monotherapy and in combination with chemotherapy [10,11,12]. Findings were similarly negative in clinical trials of pertuzumab [13,14], an inhibitor of HER2 dimerization. Clinical trials using lapatinib, a dual epidermal growth factor receptor/HER2 tyrosine kinase inhibitor, also failed to demonstrate a significant clinical response [15,16]. Several factors may account for the lack of efficacy observed in these clinical trials, which is likely due to a combination of biological and methodological factors. From a biological perspective, treatment failures may have resulted due to low levels of HER2 expression insufficient for therapeutic targeting [17], low dependency on HER2 signaling for prostate cancer cell survival, or compensatory upregulation of androgen receptor signaling following HER2 inhibition, promoting tumor survival [2,3]. From a methodological standpoint, small recruitment size, lack of patient selection based on HER2 status, and absence of consensus scoring for HER2 IHC in prostate cancer may have affected the therapeutic outcomes. Collectively these initial data indicate that targeting HER2 with antibodies or small molecules has limited efficacy in unselected prostate cancer populations.

In contrast, recent clinical trials suggest that trastuzumab deruxtecan (T-DXd), an ADC composed of trastuzumab linked to a topoisomerase inhibitor, may benefit patients with advanced prostate cancer. Unlike unconjugated HER2-targetingmonoclonal antibodies, which require relatively homogenous and widespread HER2 IHC expression to achieve clinical efficacy, T-DXd can exploit even low-levels of HER2 expression as a portal for delivering a potent cytotoxic payload with a bystander killing effect, thus exerting strong anti-tumor activity even in tumors with low HER2-expression [17], expanding the utility of HER2 targeted therapy beyond classically HER2-high tumors such as breast cancer. The DESTINY-PanTumor02 trial is a phase II, open-label, multi-center study that began in 2020 with the goal of assessing the efficacy of T-DXd in patients 18 years or older with locally advanced, unresectable, or metastatic solid organ tumors. This includes biliary tract, bladder, cervical, endometrial, ovarian and pancreatic tumors, while excluding tumors of breast, colorectal, gastric or non-small-cell lung origin. Preliminary results indicate that T-DXd can produce clinically meaningful responses across a range of solid tumors that exhibit HER2 IHC expression. Notably patients with IHC 3+ tumors scored using the gastric cancer scoring system demonstrated the highest response rates, longer durability of response, and greatest overall survival [18]. These findings helped establish HER2 as a tumor-agnostic actionable target and ultimately led to regulatory approval of T-DXd for HER2 IHC 3+ unresectable or metastatic solid tumors. Moreover, results from trials in HER2-low breast cancer [19,20] further support the clinical utility of T-DXd in solid tumors with variable and heterogeneous HER2 expression, indicating that the threshold for HER2 expression required for clinically significant response may be lower than initially expected. Collectively, these data provide a strong biological and clinical rationale for further investigating T-DXd in advanced prostate cancer.

## 4. Standardized HER2 Testing in Prostate Cancer

The lack of a standardized HER2 testing criteria for prostate cancer is a major ongoing limitation in the field of genitourinary oncology and represents a critical barrier to realizing the therapeutic potential of HER2-targeted therapies. Early efforts to characterize HER2 expression in prostate cancer highlight a fundamental discordance between HER2 protein expression and gene amplification in prostate cancer. Minner et al. carried out a large TMA study using more than 2000 prostate cancer specimens with two different antibodies for HER2 IHC and FISH [6]. They identified HER2 with IHC expression in 17.2% to 22.5% of primary prostate cancer cases, but only found *HER2* amplification in 0.04% of cases using FISH analysis. These findings differ markedly from patterns observed in breast cancer, where HER2 amplification and protein overexpression are tightly linked, underscoring fundamental biological differences in HER2 regulation across tumor types. To date, HER2 IHC scoring approaches adapted from well-established breast, gastric, and gastro-esophageal junction cancer (GEJC) guidelines have been applied to prostate cancer. However, these methods have yielded highly variable HER2 prevalence estimates, ranging from less than 5% to more than 67% across studies [3,7,21,22]. This wide disparity highlights the urgent need for standardization of HER2 testing criteria in prostate cancer that can reliably identify clinically relevant HER2 expressions that would otherwise be overlooked.

The importance of organ-specific scoring is well illustrated by GEJC tumors. HER2 assessment criteria were specifically developed for GEJC tumors to account for differences in membrane immunoreactivity and tumor heterogeneity relative to breast cancer [23]. Similarly, the spatial distribution of HER2 expression in prostate cancer is complex, displaying both intra- and intertumoral heterogeneity along with temporal changes that occur even without HER2-directed treatments. These factors likely contribute to the inconsistent HER2 prevalence reported in the literature [3,7,21,22,24]. Therefore, developing a prostate cancer-specific HER2 scoring system may be as crucial as ensuring adequate tissue sampling and appropriate biopsy timing. Establishing such standardized criteria will be essential for harmonizing HER2 assessment across studies and for advancing HER2-targeted therapies in prostate cancer.

## 5. Recent Studies Involving HER2 Expression in Prostate Cancer

A recent study by Lee et al. [25] concluded that HER2 expression is rare in metastatic prostate cancer, thus limiting its utility as a therapeutic target. Using 1 mm TMAs constructed from post-mortem tumors of 52 advanced prostate cancer patients, the authors scored HER2 expression with criteria validated for GEJC, which requires membranous immunoreactivity in clusters of at least five cohesive neoplastic cells [26]. Using this approach, most tumors were scored as 0 (74%) or 1+ (16%), five patients were scored as 2+ (10%), and none were scored as 3+, with low concordance across metastatic sites. While methodologically rigorous, these findings may underestimate true HER2 expression in prostate cancer. Small cores of tissue may fail to capture focal or heterogeneous expression patterns, and reduced immunoreactivity in older archival samples due to protein degradation may further diminish detection sensitivity [27,28]. In light of prior studies reporting HER2 staining in upward of 50% of metastatic samples [3,7,21,22], this conclusion may warrant further consideration, as it may overlook patients who could benefit from HER2-targeted therapies.

## 6. Proposed HER2 Prostate-Specific Scoring Syste

Our own evaluation of HER2 expression in a cohort of 231 patients across various stages of prostate cancer found that 58.9% expressed HER2, including 3+ in 5.2% and 2+ in 18.2% [3]. For this study, we used a modified scoring scheme adapted from GEJC criteria but omitted cell cluster requirements to effectively increase the dynamic range of HER2 expression, allowing for a more in-depth analysis of variable and low-level expression (Table 1). Importantly, higher HER2 expression using our system correlated with advanced disease, which is consistent with prior studies [6,7,29] that found an association between aggressiveness of prostate cancer and increased HER2 expression.

## 7. Clinical Trials with Antibody–Drug Conjugates Targeting HER2 in Prostate Cancer

Subsequently, we treated two patients with metastatic prostate cancer whose tumors were scored as HER2 3+ using our prostate-specific IHC scoring system with T-DXd [30,31]. Both patients demonstrated durable and clinically meaningful responses (Table 2). The first patient [30] had poorly differentiated prostate adenocarcinoma with multiple sites of metastases at the time of diagnosis. He was initially treated with androgen deprivation therapy and docetaxel, but his disease progressed. He subsequently received six additional lines of therapy, including lutetium Lu-177-PSMA-617 and cabazitaxel plus carboplatin, and participated in three clinical trials. Despite these interventions, his disease continued to progress, and he was ultimately diagnosed with treatment-emergent neuroendocrine prostate cancer (t-NEPC) with metastasis to the brain. IHC staining of both the primary tumor and a metastatic brain lesion demonstrated HER2 IHC 3+ expression using our modified scoring system. Based on these findings, treatment with T-DXd was initiated, and after 4 cycles, a 57% overall reduction in tumor volume, including a 62% reduction in brain metastasis, was achieved [30,31].

The second patient was also with metastatic prostate cancer and progressed through six prior lines of therapy [31]. HER2 IHC was performed on a rectal metastasis demonstrating HER2 IHC 3+ expression using our prostate-specific scoring criteria. The patient was treated with T-DXd in combination with abiraterone and, after six cycles, demonstrated a 70.5% reduction in tumor volume, including a 52% reduction in liver metastasis. It is important to highlight that both patients had exhausted all standard treatment options and would not have been considered eligible for treatment with T-DXd based on the existing pan-tumor approval criteria, which rely on the GEJC scoring system and are approved only for HER2 IHC 3+ solid tumors. These patients’ tumors would have been classified as either HER2 1+ (breast) or 2+ (GEJC), underscoring the potential clinical consequences of non-prostate-specific HER2 scoring. To further investigate this therapeutic approach, we are currently conducting a phase II clinical trial (NCT06610825; CaRPET) evaluating T-DXd in patients with HER2-positive metastatic castration-resistant prostate cancer.

## 8. Conclusions

In summary, this commentary highlights the need for a prostate-specific HER2 scoring system that accurately represents the HER2 expression patterns seen in prostate cancer. Such standardization is essential for ensuring appropriate IHC grading and enabling accurate identification of patients who may be eligible for HER2-targeted therapy. Our aim for this grading system is to be adopted and further validated in future trials investigating HER2 in prostate cancer. In addition, our findings support a potential therapeutic role of HER2-targeted ADC therapy in prostate cancer, particularly in patients who have failed numerous lines of therapy, based on the clinically meaningful responses seen in our two patients treated with T-DXd. Although HER2 expression is heterogeneous in prostate cancer, this does not necessarily preclude its value as a therapeutic target, as seen in HER2-low and ultra-low breast cancer. Further studies should aim to evaluate the predictive value of different levels of HER2 expression and the use of HER2-targeted ADC therapies in advanced prostate cancer.

## Figures and Tables

**Table 1:** HER2 Immunohistochemistry (IHC) scoring for breast and GEJC specimens according to the College of American Pathologists (CAP) and American Society of Clinical Oncology (ASCO) compared to the prostate-specific HER2 IHC scoring system developed in the DC Veterans Affairs Medical Center [3].

Score	Breast	GEJC Surgical Specimen	GEJC Biopsy Specimen	Prostate Biopsy and Surgical Specimen Scoring Developed in the DC VAMC
0	No staining or incomplete membrane staining ≤10% tumor cells	No staining or membranous staining in <10% of tumor cells	No staining or no membranous staining in any tumor cells	No staining in any tumor cell(s)
1+	Incomplete membrane staining that is faint/barely perceptible and within >10% tumor cells	Faint or barely perceptible partial membranous staining in ≥10% of tumor cells	Tumor cell cluster * with faint or barely perceptible membranous staining, irrespective of positive tumor cell percentage	Faint (barely perceptible) membranous staining in any tumor cell(s)
2+	Weak to moderate complete membrane staining >10% tumor cells or intense membrane staining in ≤10% tumor cells	Weak to moderate complete, basolateral or lateral membranous staining in ≥10% of tumor cells	Tumor cell cluster * with a weak to moderate complete, basolateral or lateral membranous staining irrespective of positive tumor cell percentage	Weak to moderate complete, basolateral or lateral membranous staining in any tumor cell(s)
3+	Complete membrane staining that is intense and >10% of tumor cells	Strong complete, basolateral or lateral membranous staining in ≥10% of tumor cells	Tumor cell cluster * with a strong complete basolateral or lateral membranous staining irrespective of positive tumor cell percentage	Strong complete, basolateral, or lateral membranous staining in any tumor cell(s)

GEJC: Gastroesophageal Junction Cancer. * Tumor cell cluster consists of ≥5 neoplastic cells. Due to heterogeneous and scattered and HER2 expression in prostate cancer, only intensity of membranous staining was considered for scoring, and the percentage/number of stained tumor cells was not taken into consideration.

**Table 2:** Summary of clinical responses to T-DXd in two patients with metastatic prostate cancer exhibiting HER2 IHC 3+ expression as assessed using our prostate-specific HER2 scoring system.

	Patient 1	Patient 2
Patient Information		
Age at initial diagnosis (years)	54	55
Age at treatment with T-DXd (years)	60	64
Tumor type	Prostate adenocarcinoma with treatment-emergent neuroendocrine prostate cancer	Prostate adenocarcinoma
TNM stage	Stage IV	Stage IV
HER2 IHC score by breast scoring system	1+	1+
HER2 IHC score by GEJC scoring system	2+	2+
Gleason Score	4 + 5, Grade Group 5	Unable to grade, poorly differentiated metastatic biopsy
Failed lines of therapy	Androgen deprivation therapy, docetaxel, abiraterone, high dose testosterone (BAT trial), enzalutamide, lutetium Lu 177 vipivotide tetraxetan	Leuprolide, docetaxel, sipuleucel-T, abiraterone, docetaxel, carboplatin, lutetium Lu 177 vipivotide tetraxetan, olaparib, pembrolizumab
Type of Intervention	T-DXd only for 7 months, followed by T-DXd + abiraterone	T-DXd + abiraterone
Radiographic response to T-DXd	Decrease in tumor volume by 57%	Decrease in tumor volume by 70.5%
Duration of Response with T-DXd	7 months with monotherapy and 7 months with combination therapy	7 months with combination therapy

## Data Availability

No new data were created or analyzed in this study. Data sharing is not applicable to this article.
